# Bouveret's syndrome: A rare case of gallstone causing gastric outlet obstruction

**DOI:** 10.1002/ccr3.8969

**Published:** 2024-05-30

**Authors:** Alifa Sabir, Ruqia Mushtaq, Rabia Arshad, Noor Khalid, Maheen Ayub, Shahzaib Maqbool, Muhammad Farhan, Muhammad Hanif, Abdulqadir J. Nashwan

**Affiliations:** ^1^ Department of General Surgery Benazir Bhutto Hospital Rawalpindi Pakistan; ^2^ Hamad Medical Corporation Doha Qatar

**Keywords:** Bouveret's syndrome, case report, gallstones, gastric outlet obstruction, intestinal fistula, intestinal obstruction

## Abstract

**Key Clinical Message:**

The case highlights the importance of decisive action in addressing large gallstones causing gastric outlet obstruction. The chosen single‐stage surgical approach reflects the need to manage both obstruction and the gallstone simultaneously.

**Abstract:**

Bouveret's syndrome is a rare cause of gastric outlet obstruction secondary to gallstones entering the enteric system through an acquired cholecystoduodenal fistula. Here, we present the case of an 85‐year‐old female who presented to our emergency department with gastric outlet obstruction secondary to a large gallstone in the third part of the duodenum. Abdominal X‐ray did not demonstrate air‐fluid levels but revealed a dilated gastric shadow, suggesting gastric outlet obstruction. EGD showed a dilated stomach and a hard, golf ball‐sized gallstone in the duodenum. CT scan showed a distended stomach with a large gallstone obstructing the DJ junction and air in the biliary tree. Findings were suggestive of perforation of the gallbladder with stone impaction in the duodenojejunal (DJ) junction. The patient was managed surgically with a one‐stage procedure comprising enterotomy, fistula closure, and cholecystectomy. Although Bouveret's syndrome is rare, it is important for practicing surgeons to have a high index of suspicion for this condition due to the high mortality associated with it.

## Graphical abstract

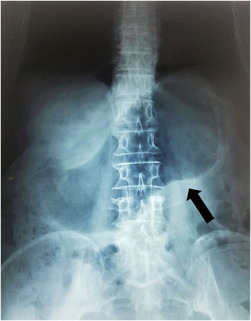

## 1 INTRODUCTION

Bouveret's syndrome is a rare gallstone ileus secondary to an acquired fistula between the gallbladder and the duodenum or stomach. Through the fistula, a stone from the gallbladder may enter the enteric system and cause intestinal obstruction.^1^ Cholelithiasis is a common gastrointestinal problem, which, although asymptomatic, can lead to several complications. Gallstones become symptomatic at the rate of 1%–4% each year, and the most common complication is cholecystitis.^2^ Gallstone ileus is a rare complication of cholelithiasis reported in 0.3%–0.5% of patients with cholelithiasis.^3^ Here, we present the case of an 85‐year‐old female with gastric outlet obstruction, later diagnosed as Bouveret's syndrome.

## 2 CASE HISTORY/EXAMINATION

An 85‐year‐old female with no known comorbid conditions presented to the emergency department with a history of epigastric pain and vomiting for 5 days. The patient had not had a bowel movement for the last 2 days. Her drug history was insignificant except for over‐the‐counter antacids use.

On general examination, the patient was tachycardic with a pulse of 93 beats per minute, BP of 90/60 mmHg, afebrile, and was mildly dehydrated. Examination of the abdomen revealed distention without significant tenderness or guarding. Bowel sounds could not be appreciated. Digital rectal examination (DRE) was unremarkable.

## 3 METHODS

### 3.1 Differential diagnosis & investigations

**FIGURE 1:**
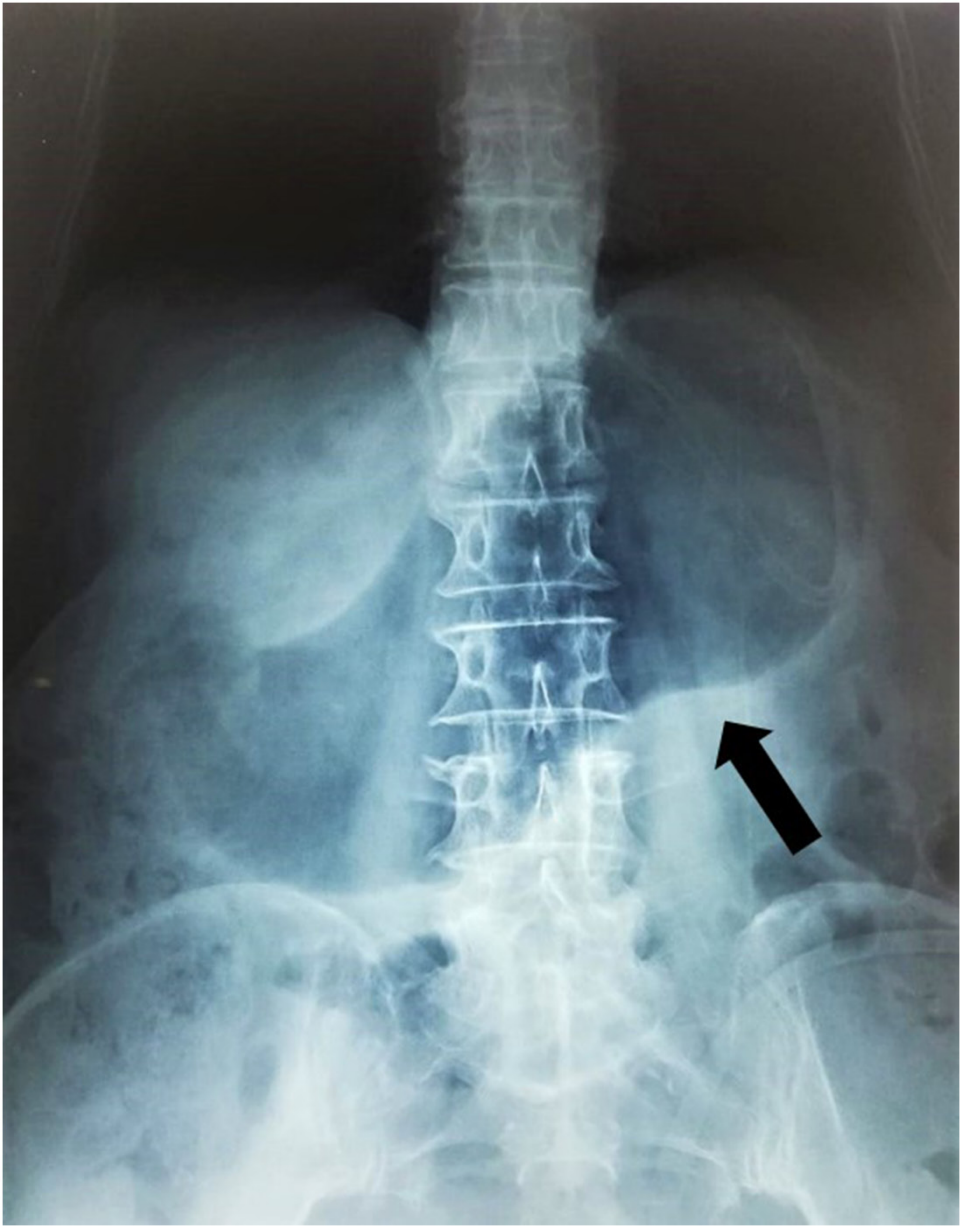
X‐ray of erect abdomen showing a dilated stomach (black arrow), displacing the small bowel inferiorly, suggestive of gastric outlet obstruction.

Baseline investigations were within reference range, expect for mild increase in ALT. Abdominal X‐ray did not demonstrate air‐fluid levels but revealed a dilated gastric shadow, suggesting gastric outlet obstruction (Figure 1). The patient was managed immediately with nasogastric intubation, intravenous hydration, and correction of her electrolyte abnormalities. A subsequent esophagogastroduodenoscopy (EGD) was planned to rule out carcinoma due to the increased age of the patient.

**FIGURE 2:**
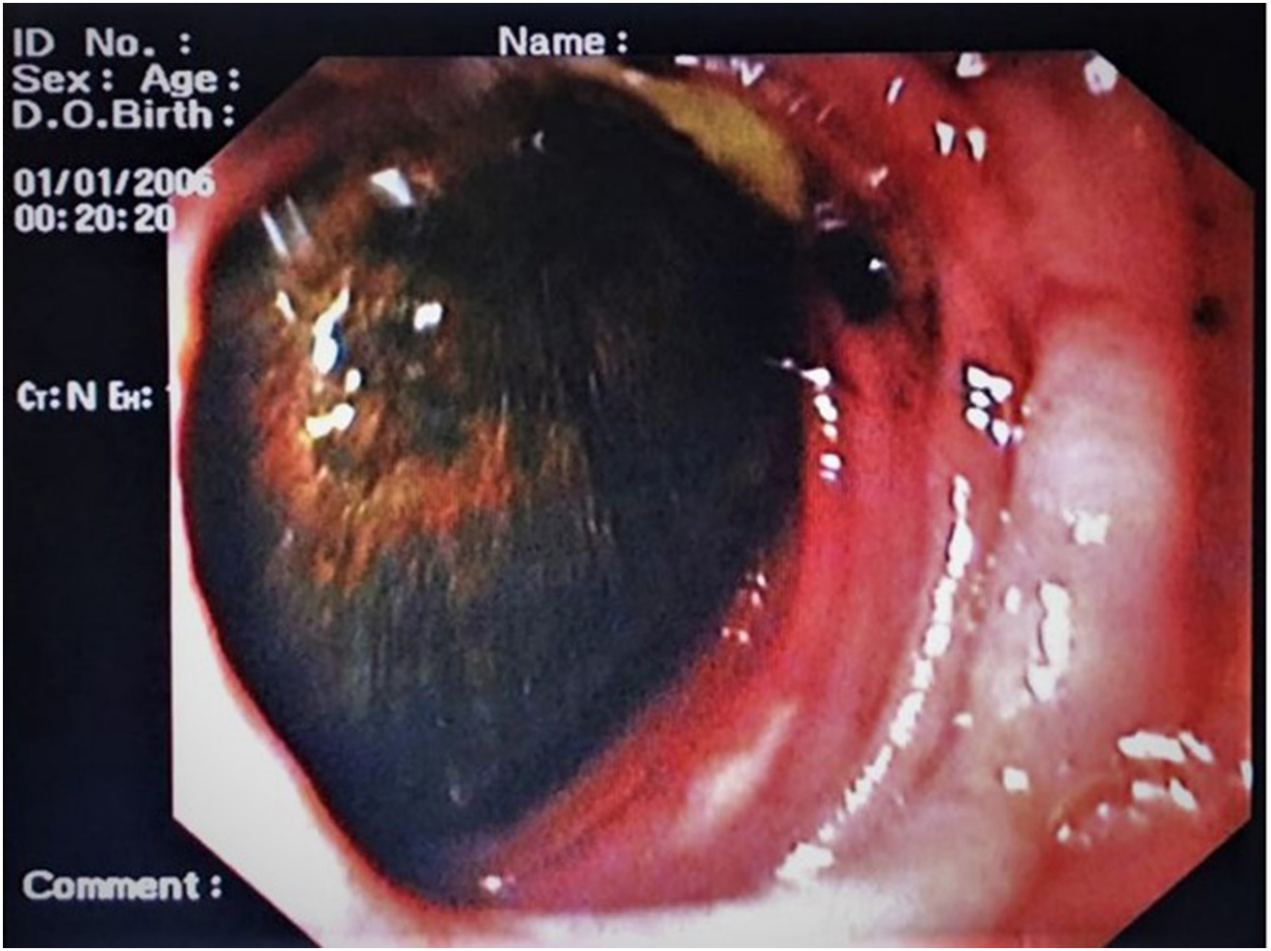
Upper gastrointestinal endoscopy showing a large gallstone in the third part of the duodenum, obstructing the further passage of the endoscope.

**FIGURE 3:**
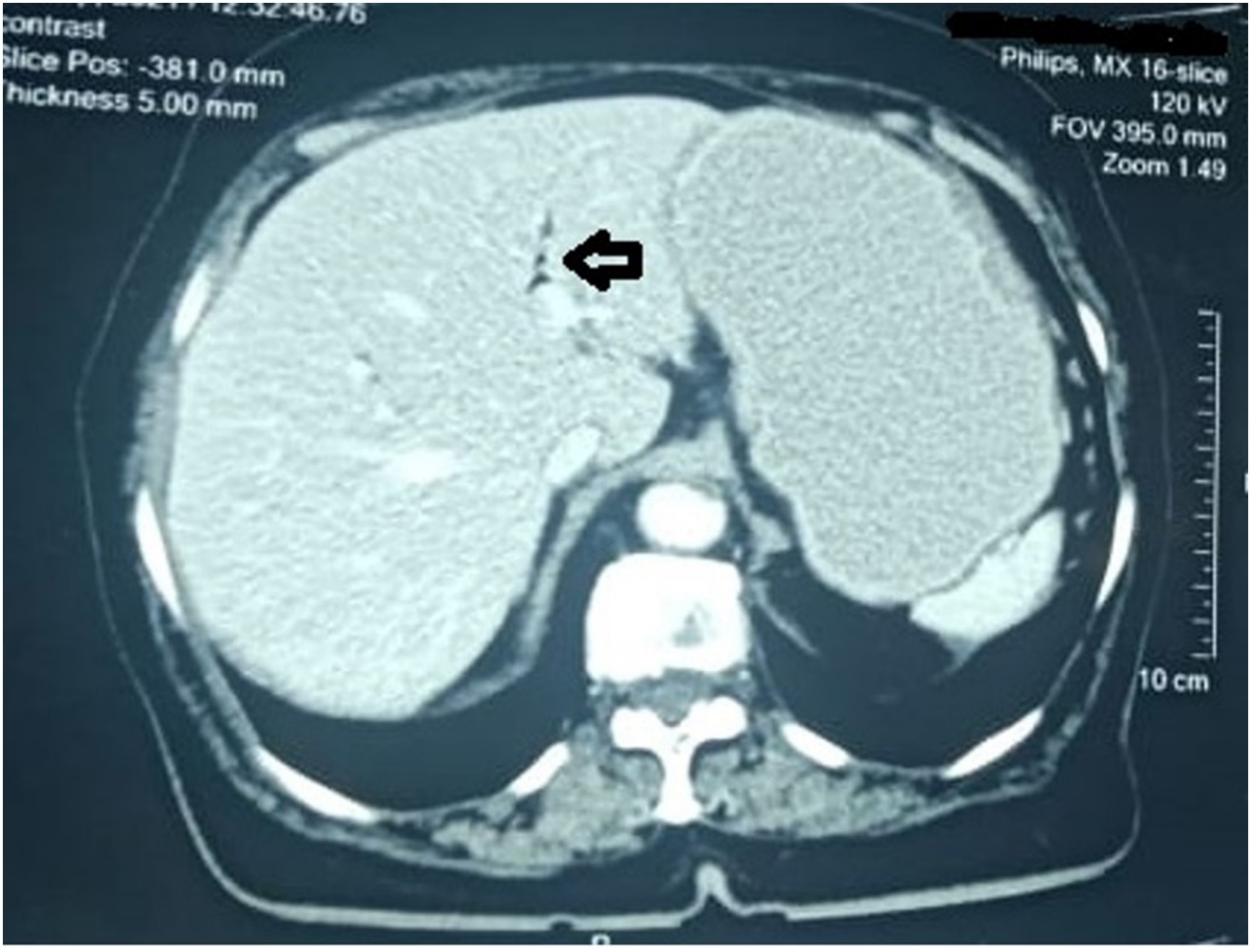
Contrast‐enhanced CT abdomen showing Rigler's triad: a distended stomach secondary to obstruction, air within the biliary channels (black arrow).

**FIGURE 4:**
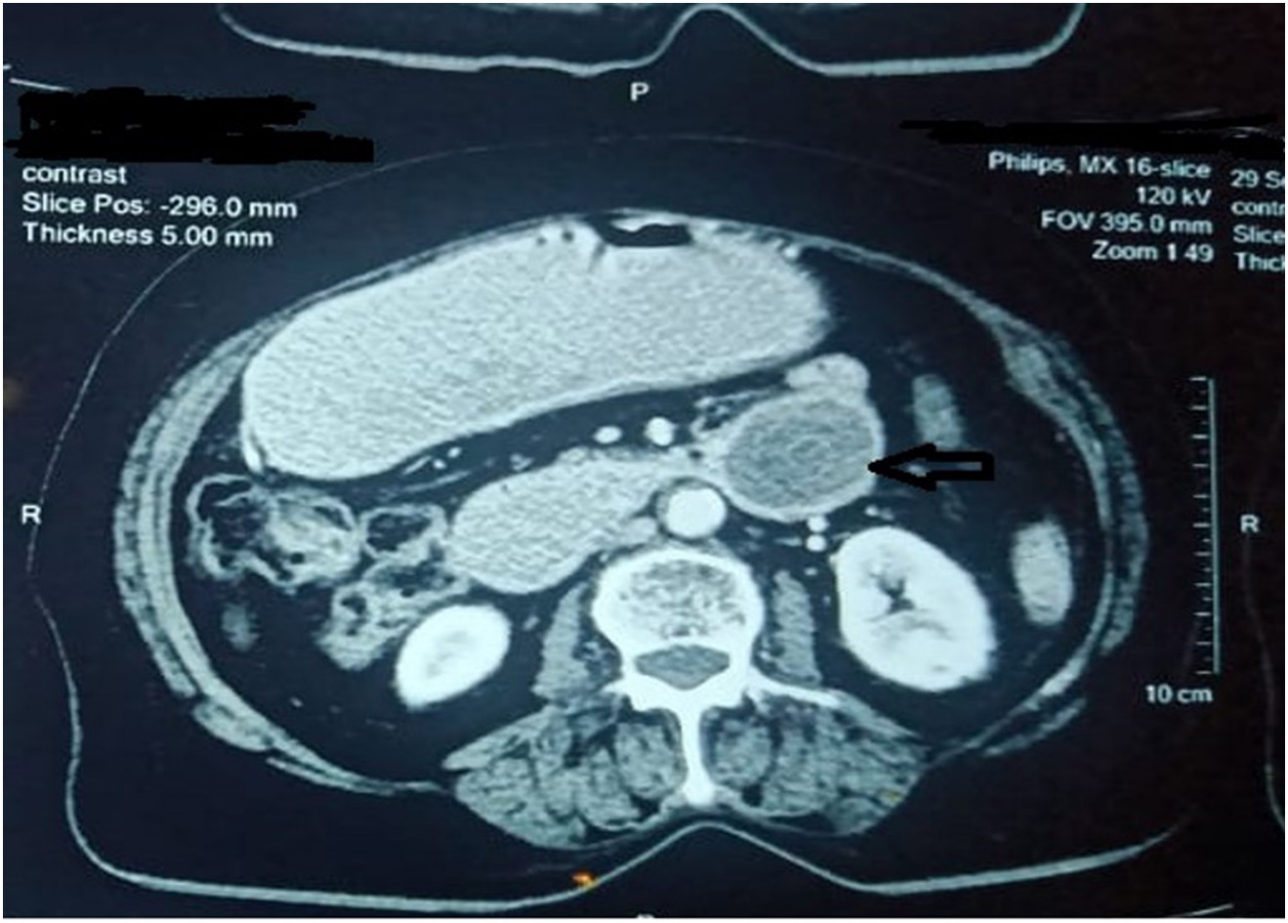
Contrast‐enhanced CT showing a large stone in a whorled appearance at the duodenojejunal junction (black arrow).

EGD showed a dilated stomach and a hard, golf ball‐sized gallstone in the duodenum (Figure 2). Spiral abdominal computed tomography (CT) scan with contrast showed a distended stomach with a large gallstone obstructing the DJ junction and air in the biliary tree. Findings were suggestive of perforation of the gallbladder with stone impaction in the duodenojejunal (DJ) junction (Figures 3 and 4).

### 3.2 Treatment

**FIGURE 5:**
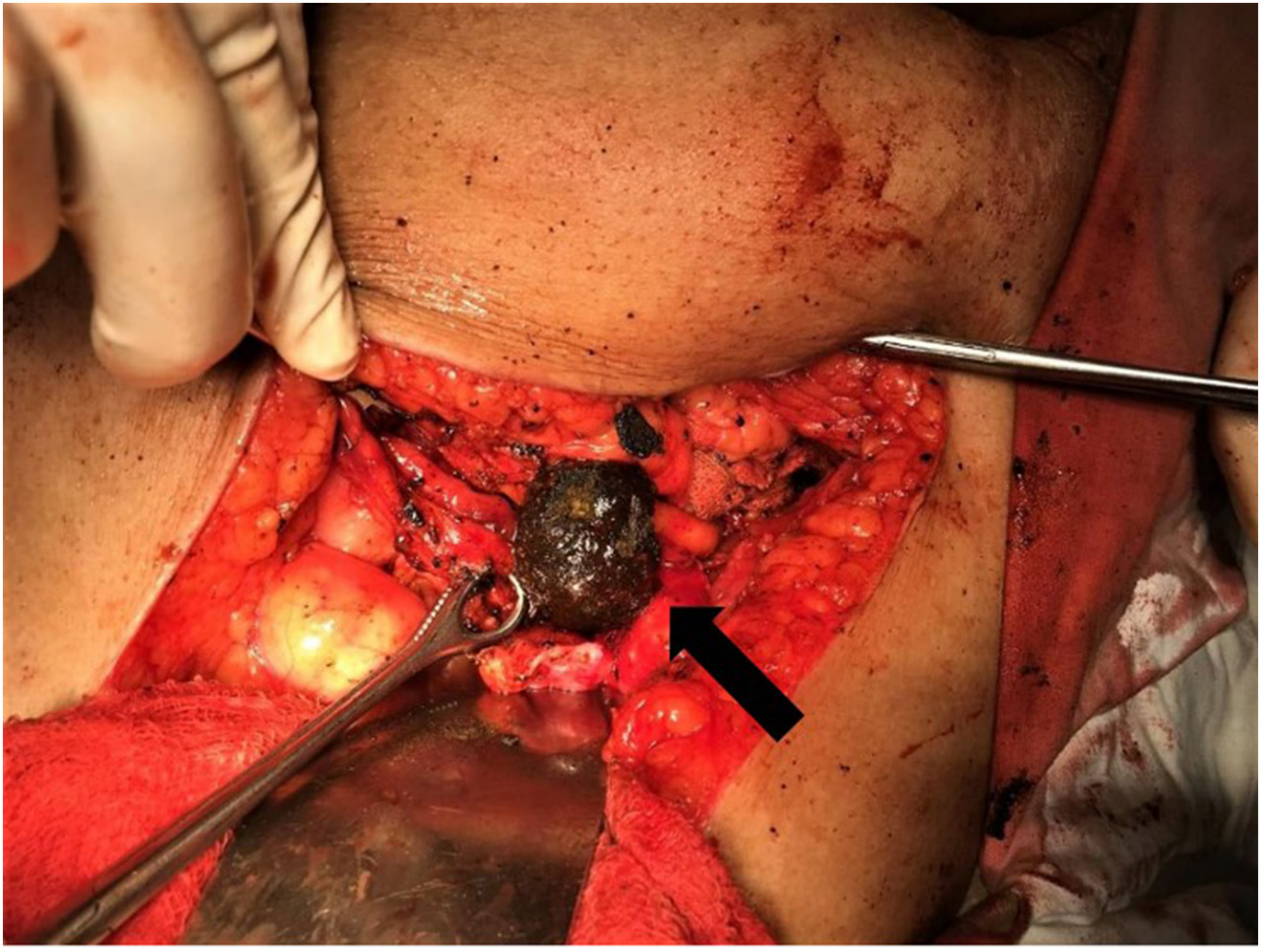
Intraoperative photograph showing gallstone retrieved from the duodenum (black arrow).

The patient was suspected of having an intestinal obstruction based on the clinical picture, and an erect X‐ray of the abdomen was obtained in the emergency. After the diagnosis of Bouveret's syndrome was made, a one‐stage surgical procedure was planned. Intraoperative findings included a thick‐walled gall bladder with surrounding inflammation and the presence of a fistulous tract with the duodenum. Enterotomy was done, and a gallstone measuring 3.7 cm in diameter was retrieved from the third part of the duodenum (Figure 5). The fistulous tract was closed, and the gallbladder was removed. Postoperative recovery was uneventful. NG tube was removed on the 1st perioperative day, allowed to be taken orally, and was well tolerated.

### 3.3 Outcome and follow‐up

The patient was discharged on the third postoperative day on analgesics and proton pump inhibitors, after which the patient was followed up initially with no reported complaints; however, they lost follow‐up after initial visits.

## 4 DISCUSSION

Cholelithiasis is a common gastrointestinal problem, which, although asymptomatic, can lead to several complications. Gallstones become symptomatic at the rate of 1%–4% each year, and the most common complication is cholecystitis.^2^ Gallstone ileus is a rare complication of cholelithiasis reported in 0.3%–0.5% of patients with cholelithiasis.^3^ It typically occurs in elderly females with multiple medical comorbidities and is associated with high morbidity and mortality.^3^

Bouveret's syndrome is an unusual type of gallstone ileus that occurs when a stone migrates from the gallbladder to either the stomach or the intestine through a bilioenteric fistula. This results in gastric outlet obstruction, especially when the stone is >2.5 cm in size.^4^ These bilioenteric fistulas (also sometimes referred to as cholecystoduodenal fistulas) occur secondary to long‐standing pericholecystic inflammation leading to adhesions between the gallbladder and stomach or duodenum, coupled with the pressure effect of stones.^1^, ^5^

The clinical presentation of Bouveret's syndrome is nonspecific, although abdominal pain, nausea, and vomiting may be present.^6^ It is mainly diagnosed on the basis of the clinical picture, the presence of pneumonia, the demonstration of duodenal obstruction, and the visualization of the stone by ultrasound.^7^ In approximately one‐third of the cases, radiographs demonstrate Rigler's triad, which includes pneumonia, dilated stomach, and an ectopic gallstone, and is considered diagnostic for the condition. Rigler's triad can also be demonstrated by ultrasound; however, it is less specific, and contrast‐enhanced CT of the abdomen is considered the best modality for diagnosis.^1^, ^3^

Treatment is directed toward the removal of obstructing stones, which can be performed via endoscopic or surgical techniques, with or without cholecystectomy and fistula repair.^8^

Endoscopically accessible impacted gallstones are amenable to less invasive alternative therapeutic options, including EHL, extracorporeal shock wave lithotripsy, extracorporeal laser lithotripsy, and endoscopic mechanical lithotripsy for fragmentation. To date, there have been a few case reports of the successful use of endoscopic techniques in patients with gallstones impacted within the stomach and small intestine. The endoscopic lithotripsy techniques allow for the fragmentation of large, calcified stones that would not otherwise be amenable to other endoscopic modalities. Advanced endoscopic skills, in addition to the proper equipment, are necessary as the technique has the potential to be long and complicated but can ultimately be successful.^9^

Surgical enterolithotomy can be done with the repair of the fistula and cholecystectomy in a single‐stage procedure, which is associated with higher morbidity. A two‐stage procedure is where cholecystectomy is performed after 4–6 weeks to allow spontaneous closure of the fistula. However, it poses a risk of further complications from gallstones, and currently, the best approach varies on a case‐to‐case basis.^1^, ^10^

The findings of our case are similar to those of previously published case reports in terms of patient presentation and diagnosis. It has been previously suggested that older patients undergo a two‐stage procedure to reduce operative risk.^4^, ^8^ However, our patient underwent a successful one‐stage procedure at the age of 85 years. No follow‐up data on the morbidity of the procedure are available, which is a limitation of our case.

## 5 CONCLUSION

Bouveret's syndrome is an uncommon presentation of gallstone ileus. the diagnosis can be confirmed by visualizing Rigler's triad on abdominal CT. A high index of suspicion is needed when patients present with bowel obstruction and a previous history of gallstones to prevent morbidity and mortality from Bouveret's syndrome. The patient was managed surgically with a one‐stage procedure comprising enterotomy, fistula closure, and cholecystectomy. Although Bouveret's syndrome is rare, it is important for practicing surgeons to have a high index of suspicion for this condition due to the high mortality associated with it.

## AUTHOR CONTRIBUTIONS

**Alifa Sabir:** Writing – original draft; writing – review and editing. **Ruqia Mushtaq:** Writing – original draft; writing – review and editing. **Rabia Arshad:** Writing – original draft; writing – review and editing. **Noor Khalid:** Writing – original draft; writing – review and editing. **Maheen Ayub:** Writing – original draft; writing – review and editing. **Shahzaib Maqbool:** Writing – original draft; writing – review and editing. **Muhammad Farhan:** Writing – original draft; writing – review and editing. **Muhammad Hanif:** Writing – original draft; writing – review and editing. **Abdulqadir J. Nashwan:** Writing – original draft; writing – review and editing.

## FUNDING INFORMATION

This study received no funding.

## CONFLICT OF INTEREST STATEMENT

The authors declare that they have no conflicts of interest.

## ETHICS APPROVAL

Ethical approval was not required for this study in accordance with local or national guidelines.

## CONSENT

Written informed consent was obtained from the patient to publish this report in accordance with the journal's patient consent policy.

## Data Availability

All data generated or analyzed in this study are included in this published article. Further inquiries can be directed to the corresponding authors.
